# Enhancing preclinical speech-language pathology students’ self-perceived clinical competence using simulated patients

**DOI:** 10.1590/2317-1782/e20250054en

**Published:** 2026-01-30

**Authors:** Estella Pui-man Ma, Taiying Lee, Wing-hong Li

**Affiliations:** 1 Programme of B.Sc. in Speech-Language Pathology, Faculty of Education, The University of Hong Kong – HKU - Hong Kong.; 2 School of Health Sciences, Western Sydney University - Penrith, Australia.; 3 School of Drama, The Hong Kong Academy for Performing Arts - Hong Kong.

**Keywords:** Speech-Language Pathology, University Students, Simulation Training, Learning, Clinical Education, Clinical Reasoning, Clinical Decision Making

## Abstract

**Purpose:**

This study aimed to evaluate the impact of preclinical simulation-based learning experience in reducing student anxiety in interacting with real patients and enhancing their self-perceived clinical competence.

**Methods:**

Second-year undergraduate speech-language pathology (SLP) students undertaking a preparatory course for clinical work and placement participated. Two clinical case simulation sessions were embedded as part of this course. In each session, two professional actors role-played as caregivers of family members with communication disorders. The first simulation focused on foundational assessment skills, and students were required to obtain a case history with the simulated caregivers. The second simulation focused on intervention, and students recommended communication strategies to the simulated caregivers. Students’ self-perceived level of skills, confidence and anxiety were assessed before and after the simulation sessions.

**Results:**

Students reported significant increases in their confidence level following simulation sessions. They perceived themselves as much better prepared for working with real patients in upcoming clinical placements.

**Conclusion:**

Simulation-based learning experience in a controlled environment enhances preclinical SLP students’ perceived confidence levels and clinical competence.

## INTRODUCTION

Promoting the transition from theory to practice has always been a prime priority in the training of speech-language pathologists. Traditionally, students learn the theoretical and clinical knowledge of communication disorders through coursework. They then apply the knowledge and skills learned from coursework to a clinical practicum where they work with real patients. However, the transition from theory to practice is not always smooth. Some students struggle in transforming their theoretical knowledge to practical application. The term “theory-practice gap” has been used to describe the struggles and challenges students encounter in clinical practice^(1-3)^. There are several reasons for the challenges in bridging the theory-practice gap: i) a lack of self-confidence; ii) a lack of effective consultation (interpersonal and communication) and noticing skills, and iii) a lack of opportunities to practice with individuals with communication disorders. Providing students with timely practice of consultative and clinical skills can promote better generalization from theory to practice. Ideally, such practice should be integrated as part of the coursework, by providing students with authentic clinical practice opportunities at designated times to supplement classroom instructions. Unfortunately, there are clear practical and logistic constraints (e.g., time, venue, accessibility) accessing patients with communication disorders at these designated timepoints.

In recent years, there has been a fast-growing body of healthcare education research on promoting student learning through the use of simulations. Different modalities of patient simulations, ranging from low- to high-fidelity and technological levels, have been used in healthcare education, including manikins, part task trainers, patient simulators, computer-based programs using virtual reality and augmented reality, and simulated patients^(4)^. In speech-language pathology, live simulation using simulated patients is a popular teaching and learning tool^(5)^. Simulated patients refer to individuals who do not have a health or communication condition, but they role-play to be a patient by simulating the physical appearance, symptoms, needs and perspectives of the given health problems^(4)^. They are trained to behave like real patients by reproducing the clinical symptoms of a given disorder or health condition. The term “simulated patient” is similar to “standardized patient” and has been used interchangeably in many studies, yet the two terms are technically different. Standardized patients are required to reproduce the clinical symptoms with a high level of consistency across examiners. Hence, in performance-based clinical assessment (e.g., Objective Structured Clinical Examination, OSCE) where consistency of the simulation is crucial for fair evaluation of the clinical skills competencies of healthcare students, standardized patients are used.

A typical simulation-based learning session comprises three parts: prebriefing, simulation, and post-simulation debriefing. In the prebriefing, the case scenario is introduced, together with an open discussion on the learning outcomes and the expectations from students. In the simulation session, students work with the simulated patient as if they are working with a real patient. They obtain case history information, carry out clinical examinations, or prescribe treatment with the simulated patient, depending on the learning objective of the session. Debriefing immediately follows the simulation session, in which students are guided by the simulated patients and the observing clinical educators to reflect on their performance in the session. The reflective discussion drives students’ learning through an evaluation of what went well in the session, what needs to be changed, how to change, as well as a better understanding of their own strengths and weaknesses. There are a number of advantages of using simulated patients in practicing consultation and clinical skills. The simulated patient and case scenario can be strategically designed to promote specific learning outcomes. For example, the simulated patient can portray someone who is not compliant with the clinician’s instructions, or with depression who suddenly breaks into tears in the middle of the case history taking session, or with a medically-complex condition. These simulated learning experiences provide students with opportunities to practice and learn core clinical skills as well as how to manage challenging situations or behaviors, before they see real patients in a clinical context. Moreover, students can apply clinical skills in a controlled, “risk-free” and safe learning environment.

Simulated patients have been applied successfully in developing clinical competencies in physiotherapy students^(6)^, nursing students^(7)^, and medical students. In speech-language pathology, simulated patients have been used for training and assessing students in a range of courses such as aphasia^(8-10)^, fluency disorders^(11,12)^, swallowing disorders^(13,14)^ and voice disorders^(15)^. Incorporating simulated patients in clinical training has been regularly reported by students as encouraging and rewarding learning experiences. Students reported feeling significantly more comfortable, significant increase in confidence levels and significant decrease in perceived anxiety interacting with patients after simulation. Impacts of simulation-based learning experiences do not appear to be restricted by the mode of implementation. Similar positive outcomes were reported whether the simulation was delivered in-person face-to-face or online through telepractice^(13,16)^. Different speech-language pathology boards recognize the educational value of simulation in developing students’ clinical competence. Simulation is now recognized as a formal learning experience in the clinical training of SLPs in Australia, Canada and the United States, and as an approved method in contributing to students’ direct clinical practice hours.

Early introduction of opportunities to interact and practice with patients in preclinic years, provided through simulations, can help students develop their clinical competency and enhance their confidence for the later clinical placements. Most importantly, it allows students to reflect on what and how to better equip themselves for working with real patients in clinical settings. Nevertheless, most of the evidence on simulations have been in the health education literature for nursing education and students^(7,17)^. There is a need for more research on the effectiveness and impacts of simulations for the SLP profession. The aim of this study was to evaluate the effectiveness of preclinical simulation-based learning experiences in reducing students’ anxiety with interacting with real patients and in enhancing their self-perceived clinical competence.

## METHODS

This study received approval from the Human Research Ethics Committee at The University of Hong Kong (reference number EA210276). All participants provided informed consent after they were informed about the purpose of the study, the rights, potential benefits and risks of their participation.

### Participants

Participants were second-year undergraduate students enrolled in a 5-year undergraduate speech-language pathology program at the University of Hong Kong. Students in this undergraduate program start their clinical placements in the third-year of study. None of the participating students had any prior clinical experience, and none of the students had any prior experience in simulation-based learning activities.

### Procedures

The study was a pretest-posttest experimental design. Clinical simulations were embedded as part of the course “Introduction to Clinical Practice I”. The course aims to equip students with the foundation knowledge required to succeed in clinical practice. It comprises 10 three-hour sessions which combine lecture and interactive practical activities. The course covers topics on assessment and intervention; interviewing caregivers and case-history taking, test administration, goal setting, effective communication and interaction skills, the therapeutic process, the principles of ethical practice, the clinical and supervisory processes, and Competency-Based Occupational Standards for Speech Pathologists (CBOS)^(18)^. Table 1 shows the structure of the course. Two clinical simulation sessions were embedded into the course. The first simulation session (Session 6) had an assessment focus and the second session (Session 10) had an intervention focus. The purpose of the simulation sessions was to complement the lectures and to promote students’ application of their theoretical knowledge learned from lectures to practice.

**Table 1:** Structure of the introductory level course to clinical practice

**Session**	**Mode**	**Content**
1	Lecture	Course outline and introduction
		Developing professional identify
		● Role of speech language pathologist
		● Standards of Practice and Code of ethics
		● Clinical learning and supervision in clinic placements
		● Professional conduct and communication
2 & 3	Lecture	Assessments with children
		● What is involved in assessment
		● Getting relevant clinical information from family and caregivers
		● Different types of assessments and how to use them
		● Planning assessment sessions
		● Assessment considerations
		● Communicating assessment results to family and professionals
4	Lecture	Assessment with adults
		● Tips to communicate (or to start a conversation) with adults: Qualities of a good communicator/clinician
5	Lecture	Clinical skills in professional interaction to elicit responses from adult clients (e.g., How to hold a conversation / take case history taking / instruct the adult clients for assessments and treatment / provide feedback to adult clients)
6	Simulation	Assessment focus: Case history taking with caregivers.
7 & 8	Lecture	Intervention with children
		● What to do with assessment information
		● Setting goals based on assessments
		● Intervention principles and framework
		● Key therapy strategies to work on goals in clinical sessions
		● Behaviour management strategies
		● Working with families, teachers and other professionals
		● Intervention planning and management
		● Communicating management plans and outcomes
9	Lecture	Intervention with adults
		Case studies of adult clients interacting with parents of children: Tips to talk with a parent with (i) information seeking and (ii) counselling (reassuring)
		Rationales behind general therapeutic processes with adults
10	Simulation	Intervention focus: Assessment dissemination and communication strategies to caregivers

Two case scenarios were developed for the simulation sessions (see Table 2): Case one described a child with suspected language delay. Case two described an older lady who suffered from aphasia and a swallowing disorder following a stroke. Two professional actors were recruited to be simulated caregivers, and they role-played as the child’s mother and the older lady’s daughter, respectively.

**Table 2:** The two case scenarios used in the simulation sessions

	**Case One**	**Case Two**
Background of the simulated caregiver:	A distressed young mother (28-year-old), whose 3;6-year-old boy (pseudo name: JJ) has suspected language delay.	The patient’s daughter (around 40-year-old).
	● Highly educated.	● Very busy at work, and wants to get the case history session done quickly.
	● Has just returned to Hong Kong after completing her doctoral degree at an elite university overseas.	
		● Does not get along well with her brother. Poor family dynamics.
	● Her husband is still overseas, not sure when he can be back because of flight restrictions due to the COVID-19 outbreak.	
		● Neither of them have time to take care of their mother nor to do rehabilitation exercises at home with their mother.
	● Because JJ is her first child, she is very worried about JJ’s language problems. She always compares JJ against his cousins who are the same age.	
Background of the case:	Education History	Family history
	● Currently studying at an international kindergarten.	● Widowed.
		● Live alone with a newly hired (1 month) Filipino domestic helper who speaks fair Cantonese.
	Medical History	Medical history
	● Premature.	● Suffered from aphasia and swallowing difficulties after stroke six months ago.
		● Other diseases: hypertension, diabetes mellitus.
Pre-set challenges for students:	● Feels unsure why she is being asked the long list of questions.	● Not happy with the many questions that the student clinician asks in the session.
	● Gets agitated easily during case history taking.	
		● Gets agitated easily particularly when the student asks sensitive questions (e.g., on topics around family dynamic and relationship, who provides main support for the patient, the changes since her mom had stroke).
	● When the student clinician suggests an intervention approach, she questions and counter-argues with those scientific evidence she looked up from the literature.	

The prebriefing was conducted one week before the simulation experience. Students received the case scenarios together with the expected learning objectives and task instructions. They then had a week to work on the case in small groups and to prepare for the simulation experience. During the simulation experience, students worked in groups of six to seven, with a clinical educator present. Students within the group took turns to engage with the simulated caretakers. Debriefing was conducted immediately after the simulation experience. Feedback from both the simulated caregivers and clinical educator was provided. Simulated caregivers provided constructive feedback on the clinical interaction and overall session for the students. They commented on whether they felt the interactions were comfortable, whether they felt like the student clinician had established rapport; whether they were able to understand everything the clinician said to them, and if any professional jargon was used. Clinical educators provided feedback on the students’ clinical skills competence. The post-simulation debriefing also encompassed a reflective component, where students were guided with reflection on what went well, what could be improved, and their learning from the simulation experience. Apart from the debriefing within the group, students were also provided with group feedback on any common observations across the groups, as part of the post-simulation debriefing.

Students attended two simulation sessions with different learning objectives, with one session focused on assessment and the second one on intervention. Each simulation session comprised 20 minutes of students-simulated patient interaction and 10 minutes debriefing. In the first session which was scheduled in week six of the course, students practiced foundational skills in engagement and gathering a thorough case history with the simulated caregiver. In the second session which was scheduled in week ten of the course, students were tasked to explain the communication difficulties the child/adult may encounter in everyday contexts and introduce at least one appropriate language facilitative or communication strategy to the simulated caregivers.

Students completed a questionnaire immediately before and after each simulation session. The questionnaire items were selected and adapted from the tools used in Hill et al.^(19)^ and Wilson et al.^(20)^, with minor amendments and additions based on a review of the literature related to the use of simulation in speech-language pathology education. The questionnaire contained a combination of items where responses were ranked on a 5-point Likert scale from 0 to 4 with 0 “not sufficient / not useful” and 4 “extremely sufficient / extremely useful”, and open-ended questions. Students were asked to reflect on the simulation-based learning experience with three open-ended questions: “Which part(s) of the simulated learning activity that you like the most?”, “Which part(s) of the simulated learning activity that need(s) modification?” and “In what ways do you think simulated sessions have helped you transferred the skills learned from classroom to clinic?”

## RESULTS

### Students’ self-perceived level of clinical skills, confidence and anxiety

Table 3 shows the mean, median and standard deviations of students’ self-perceived level of clinical skills, confidence, and anxiety before and after each simulation session. The responses were ranked on a Likert scale from 0 to 4, higher scores suggested better clinical skills and higher level of confidence but lower level of anxiety. Paired *t*-tests were used to compare students’ perception before and after interacting with simulation patients. Data normality assumption was examined using the Shapiro-Wilk test. All measures were confirmed to be normality distributed (all *p* > 0.05).

**Table 3:** Students’ self-perceived level of clinical skills, confidence level and anxiety levels before and after interacting with simulated patients

**Area**	**Pre-simulation**			**Post-simulation**			p-level
	**Median**	**Mean**	**(SD)**	**Median**	**Mean**	**(SD)**	
Collecting case history							
Perceived clinical skills	2	1.77	(0.79)	3	2.87	(0.58)	0.0001^*^
Level of confidence	2	1.72	(0.71)	3	2.67	(0.56)	0.0001*
Level of anxiety	3	2.68	(0.84)	1	1.59	(0.75)	0.0001*
Explaining intervention plan							
Perceived clinical skills	2	1.67	(0.85)	3	2.67	(0.61)	0.0001*
Level of confidence	2	1.60	(0.83)	3	2.72	(0.59)	0.0001*
Level of anxiety	3	2.98	(0.81)	2	1.74	(0.79)	0.0001*

* *p*-levels significant at 0.001 level are marked with an asterisk.

Responses were ranked on scale of 0 to 4, where 0 = not sufficient / confident / anxious at all whereas 4 = extremely sufficient / confident / anxious.

After each simulation session, students perceived significant increases in their clinical skills and level of confidence (*p*-levels = 0.0001). They also reported significant decreases in the level of anxiety working with real patients in the future (*p* = 0.0001). In general, students considered the simulated session experience useful in reinforcing the skills learned in class sessions (mean score = 3.13, range = 1 to 4; possible maximum = 4), as well as useful in bridging theory learned in lectures to clinical practice (mean score = 3.03, range = 2 to 4; possible maximum = 4).

### Student’s perception of the simulation-based learning experiences

In general, positive learning experiences were obtained from students. As shown in Table 4, those items related to the debriefing process received higher ratings from students. Students particularly appreciate that the interaction with simulated patients allows them to reflect on their strengths and weaknesses, so that they know the skills or attitude to improve (28.2% of students chose “strongly agree”). They also found the feedback from the simulated patients and clinical educators useful for their learning (33.3% and 35.9% of students chose “strongly agree”, respectively).

**Table 4:** Students’ ratings of their simulation learning experience (*N* = 39)

**Items**	**Strongly disagree**		**Disagree**		**Neutral**		**Agree**		**Strongly agree**	
**As a result of interaction with simulated patients (SPs):**										
^*^ I have learned a new skill.	1	(2.6%)	0	(0.0%)	4	(10.3%)	**26**	**(66.7%)**	8	(20.5%)
My interviewing skills have improved.	0	(0.0%)	2	(5.1%)	1	(2.6%)	**33**	**(84.6%)**	3	(7.7%)
My skills in providing appropriate information have improved.	1	(2.6%)	1	(2.6%)	3	(7.7%)	**34**	**(87.2%)**	0	(0.0%)
My confidence to interact with real clients in the future has increased.	1	(2.6%)	0	(0.0%)	3	(7.7%)	**33**	**(84.6%)**	2	(5.1%)
I feel less anxious about working with real clients in the future.	2	(5.1%)	1	(2.6%)	2	(5.1%)	**34**	**(87.2%)**	0	(0.0%)
Developed my interest in working with clients with communication disorders.	0	(0.0%)	1	(2.6%)	4	(10.3%)	**28**	**(71.8%)**	6	(15.4%)
* Allow me to reflect on my strengths and weaknesses, so I know the skills or attitudes to improve.	0	(0.0%)	1	(2.6%)	2	(5.1%)	**25**	**(64.1%)**	11	(28.2%)
* I enjoyed the simulated sessions.	1	(2.6%)	0	(0.0%)	3	(7.7%)	**25**	**(64.1%)**	10	(25.6%)
**About the simulation learning experiences:**										
The SP’s portrayal as caretakers of their family members with communication disorders was realistic.	0	(0.0%)	0	(0.0%)	4	(10.3%)	**26**	**(66.7%)**	9	(23.1%)
The content of the case scenarios was realistic.	0	(0.0%)	0	(0.0%)	6	(15.4%)	**25**	**(64.1%)**	8	(20.5%)
* I find the feedback from the SPs useful for my learning.	0	(0.0%)	0	(0.0%)	3	(7.7%)	**23**	**(59.0%)**	13	(33.3%)
* I find the feedback from clinical educators useful for my learning.	0	(0.0%)	0	(0.0%)	3	(7.7%)	**22**	**(56.4%)**	14	(35.9%)
* The clinical educator was helpful in facilitating the encounter with SPs.	0	(0.0%)	0	(0.0%)	7	(17.9%)	**21**	**(53.8%)**	11	(28.2%)
I was given sufficient background information about working with the SPs.	0	(0.0%)	1	(2.6%)	5	(12.8%)	**26**	**(66.7%)**	7	(17.9%)
I felt prepared for the interactions with SP.	1	(2.6%)	4	(10.3%)	8	(20.6%)	**25**	**(64.1%)**	1	(2.6%)

* The top three items with the highest percentage of students selected “strongly agree” are marked with an asterisk.

Note: Figures in bold typeface = the most chosen category by students.

### Qualitative comments obtained from open-ended questions

Responses from the open-ended questions in the post-simulation questionnaire indicated that students recognized the value and clinical relevance of the simulation experience to their clinical knowledge and skills development. Students reportedly enjoyed the learning experience and found the experience rewarding. Regarding what aspect of the simulation activity the students liked the most, their replies fell into two main categories. Over half of the students (51.3%, 20 out of 39) commended on the interactions with the simulated patients. For example, “I like how the caregivers react differently which helps us learn how to deal with sudden situations.” Another 38.4% (15 out of 39) reported they liked the debriefing session and receiving direct feedback from simulated patient as well as the clinical educator the most. Regarding any modification of the simulated learning activity to enhance student learning, one-third (13 out of 39) of the class considered no modifications were needed, 28.2% (11 out of 39) suggested that a longer session time and more variety of case types could be added. All students (100%) considered the simulation learning activity valuable for their clinical learning and requested to have simulated sessions as part of regular practice in the program curriculum.

## DISCUSSION

Promoting smooth integration of theoretical knowledge into clinical practice has long been an objective in the clinical education of speech-language pathology (SLP) students, as with other allied health and medical disciplines. In recent years, simulation-based learning has become an increasingly popular educational tool in training SLP students, and to facilitate the transfer of theoretical knowledge into clinical practice. The aim of this study was to evaluate the effectiveness of simulation experiences in enhancing pre-clinical students’ self-perceived clinical competence and confidence in interacting with real patients. Results revealed that the learning experience was in general very positive for students. Students reported a significant increase in their perceived clinical skills and confidence level, as well as a significant decrease in anxiety levels in working with real caregivers in the clinic context in the future. The present findings are consistent with the existing literature^(19,21,22)^, that healthcare clinical simulation is effective in enhancing students’ perception of clinical competency.

This group of students have no prior experience in clinical placement. They may feel more uncertain and more worried about the actual encounter with “real patients”. As revealed from the post-simulation survey, not only do students feel more confident with interacting with real patients, they are less anxious. These two perceptions are closely related but they tap into different aspects, as even students who are confident with their skills can feel anxious. The simulated environment facilitates students’ confidence in trying out the clinical skills they have been learning in lectures. The reduction in anxiety may be attributed to the debrief cycle as the students now have a clearer picture and focus on what to do in the clinic, how to improve as well as clearer ideas on their own strengths and areas needing improvement. Working with healthy actors who role-played as simulated patients provided students with a controlled, safe and supportive learning environment to “take risk” without the fear of making mistakes and to practice their clinical skills. Being more open to “take risk” especially for students at the pre-clinical stage is the key to their learning, that it is not possible to promote in real clinical settings.

The present findings provide further supporting evidence to the use of patient simulations in the training and development of foundation clinical skills in SLP students^(23,24)^. Overall, students considered the simulation-based learning experiences encouraging and rewarding. Students highly valued the debriefing component of the simulation session, as reflected from their ratings of their simulation learning experience. In the debriefing session, students receive “double feedback” – feedback on their clinical skills from the clinical educator, as well as the focused and deliberate feedback from simulated patients, which provides another view of the student’s clinical skills and attitude. The kind of feedback from the simulated patient to student clinician is not possible in the standard clinical placement. It enables the students to better reflect upon their strengths and weaknesses. Previous studies in speech-language pathology and allied health disciplines also reported debriefing as an important component of simulation-based learning and it provides students with the most learning^(25,26)^.

### Limitations and directions for future research

The following limitations should be acknowledged when interpreting the results. First, the study did not include a control group for comparison. It would be interesting to compare students’ perception between those who receive simulation sessions and those without simulation sessions, or receive other forms of clinical simulation. Second, because the researcher was involved in teaching the participants, the potential observer bias cannot be ignored. Another limitation relates to the reliance on self-perceived data rather than objective indicators of clinical competence. There should be longitudinal tracking and monitoring of students’ clinical skills performance and competence as they progress from pre-clinical to clinical years working with real patients in clinical placements. Finally, even though most students appreciated and requested for more simulation sessions, different students have different learning styles and needs. Future studies should explore the effect of learning styles on the effectiveness of simulation-based learning. It should also be highlighted that although this type of teaching methodology has proven to be efficient in reducing students’ anxiety levels and raising their self-confidence, this model should not, in any way, replace entirely actual clinical practice with real patients.

## CONCLUSIONS

Providing simulation-based learning experience in a controlled environment for preclinical SLP students is efficient in reducing students’ perceived anxiety levels and raising their self-confidence and sense of clinical readiness. Further development of clinical simulation within the SLP curriculum is much needed to inform stakeholders the best practice of simulation-based education of SLPs. Research should be conducted on examining the optimal timing, format and dosage of simulation implementation to promote student learning.
